# Saved by My Specs: Incidental central retinal vein occlusion uncovering infective endocarditis – A case report

**DOI:** 10.1016/j.idcr.2025.e02484

**Published:** 2025-12-26

**Authors:** Daniel Broderick, Ciara Murray, Padraig McGettrick, James Woo, Emer Kilbride, James McCarthy, Yvonne O’Meara, Varisha Shahzad, Carlos Mejia-Chew

**Affiliations:** a Department of Infectious Diseases, Mater Misericordiae University Hospital, Eccles Street, Phibsboro, Dublin 7, D07R2WY, Ireland; b Department of Cardiothoracic Surgery, Mater Misericordiae University Hospital, Eccles Street, Phibsboro, Dublin 7, D07R2WY, Ireland; c Department of Renal Medicine, Mater Misericordiae University Hospital, Eccles Street, Phibsborough, Dublin 7, D07R2WY, Ireland; d University College Dublin, Belfield D04 V1W8, Ireland

**Keywords:** *Granulicatella adiacens*, Infectious endocarditis, Prosthetic valve endocarditis, Nutritionally variant streptococci

## Abstract

We describe a case of a 74-year-old man with a history of bioprosthetic aortic valve replacement who was diagnosed with *Granulicatella adiacens (G. adiacens)* infective endocarditis (IE) following an incidental finding of central retinal vein occlusion (CRVO) during a routine optician visit. Despite minimal symptoms on presentation, blood cultures grew *G. adiacens*, and imaging revealed a 1 × 1.2 cm aortic valve vegetation plus splenic and cerebral embolic complications. Management was complicated by drug-induced drug rash with eosinophilia and systemic symptoms (DRESS) syndrome, microangiopathic thrombocytopenia, anaemia, and possible subacute glomerulonephritis, leading to deferral of surgery until haematological parameters improved. Following a 9-week antibiotic course and stabilisation of platelet counts, he underwent a successful redo aortic valve replacement, highlighting the indolent yet clinically significant nature of *G. adiacens* IE and the importance of thorough, multidisciplinary care in complex prosthetic valve infections.

## Introduction

*Granulicatella adiacens* (*G. adiacens*) is a fastidious, Gram-positive coccus formerly part of the nutritionally variant streptococci (NVS), but is now classified under the *Abiotrophia* and *Granulicatella* genera. It is a commensal organism in the oral cavity, gastrointestinal tract, and urogenital system, but is also recognised as a rare cause of infective endocarditis (IE) [1], [2], [3]. It can present in an indolent fashion and is often difficult to isolate by standard culture methods. This can delay diagnosis and increase morbidity and mortality [2], [3], [4]. Cases of *G. adiacens* IE remain relatively limited in the literature compared to more common organisms causing IE, such as *Staphylococcus aureus* or viridians group streptococci (VGS) [2], [3].

This report describes an unusual presentation of *G. adiacens* IE and highlights the indolent nature and widespread effects of *G. adiacens* endocarditis. It also explores the challenges of managing clinical uncertainty and surgical timing in PVE, particularly in the context of a large valve vegetation with embolic phenomena, and persistent pyrexia.

## Case Summary

**Fig. 1:**
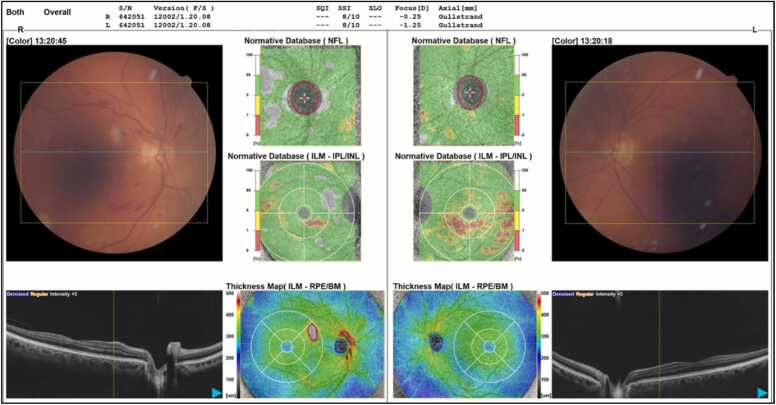
*Optical coherence tomography and fundoscopy*.

A 74-year-old man presented to his optician for a routine change of glasses. His vision was largely unchanged from two years prior, however, flame haemorrhages and cotton wool spots were noted in the right eye on fundus examination. The patient was referred to the hospital’s eye emergency department for more in-depth assessment. On arrival, he underwent further slit lamp examination and flame haemorrhages were observed in all four quadrants of the retina, as well as cotton wool spots. An optical coherence tomography (OCT) showed no macular oedema (Fig. 1). His vision, intraocular pressure, lids, conjunctiva, and cornea were all normal. The findings were consistent with a non-ischaemic central retinal vein occlusion (CRVO), which was deemed suitable for outpatient management.

However, blood test revealed a raised creatinine level (112 µmol/L; reference range: 65–107 µmol/L), elevated C-reactive protein (CRP, 56 mg/L; reference range: <7 mg/L), anaemia (haemoglobin 7.7 g/dL; reference range: 13–18 g/dL), thrombocytopenia (platelets 96 × 10⁹/L; reference range: 150–400 × 10⁹/L), and hypalbuminaemia (albumin 31 g/L; reference range: 35–50 g/L), which prompted an admission for further evaluation. On further history, he reported exertional dyspnoea without chest pain or orthopnoea for the past six-months. He also experienced two episodes of drenching night sweats over the preceding six weeks. His past medical history was remarkable for bioprosthetic aortic valve replacement seven years earlier, varicose veins, previous lower limb cellulitis, hypertension, and an ascending aortic aneurysm (4.5 cm, under surveillance). There was no recent history of travel, joint pain, swellings, or rashes. The patient was a retired plasterer and an ex-smoker with a 30 pack-year history. He also had a significant alcohol history, having consumed approximately 60 units per week until six months before admission. He remained independent in all activities of daily living at baseline. Notably, three weeks prior to admission, he had undergone a dental procedure that resulted in an odontogenic infection requiring antibiotic treatment.

On examination, he had an end-systolic murmur radiating to the carotids, grey finger discolouration, bilateral pedal oedema, and petechiae of the lower limbs. Initial bedside investigations included a urine dipstick, which showed 2 + blood and 1 + protein, and a chest X-ray demonstrating a left lower zone opacity. A transthoracic echocardiogram (TTE) showed moderate mitral valve regurgitation but no vegetations or aortic valve dysfunction. On the third day of admission, he developed a cough and fever (38.0 °C). His CRP increased to 61 mg/L and he complained of worsening shortness of breath. Blood cultures were sent and later grew *G. adiacens*. Intravenous (IV) ceftriaxone and vancomycin were initiated. Repeat blood cultures 24 h after commencing antimicrobials were negative.

A transoesophageal echocardiogram (TOE) showed a large vegetation on the bioprosthetic aortic valve. This finding was also seen in a CT thorax, abdomen, and pelvis (CT TAP), which demonstrated a 1 × 1.2 cm vegetation on the anterior aspects of the left and right metallic cusps of the implanted aortic valve, along with splenomegaly and multifocal splenic infarcts, likely due to septic emboli. Given that he fulfilled major Duke criteria for IE, care was transferred to the Infectious Diseases (ID) team, and gentamicin was added to his antimicrobial regimen. As there are currently no EUCAST-defined antimicrobial susceptibility breakpoints for *G. adiacens*, interpretation of Minimal inhibitory concentrations (MIC) required extrapolation from Clinical and Laboratory Standards Institute (CLSI) guidelines: the MIC for penicillin was 0.125 μg/ml and for vancomycin was 0.75 μg/ml (<2.0). Hence, vancomycin was discontinued on day 8 of admission and gentamicin continued (Fig. 4).

**Fig. 2:**
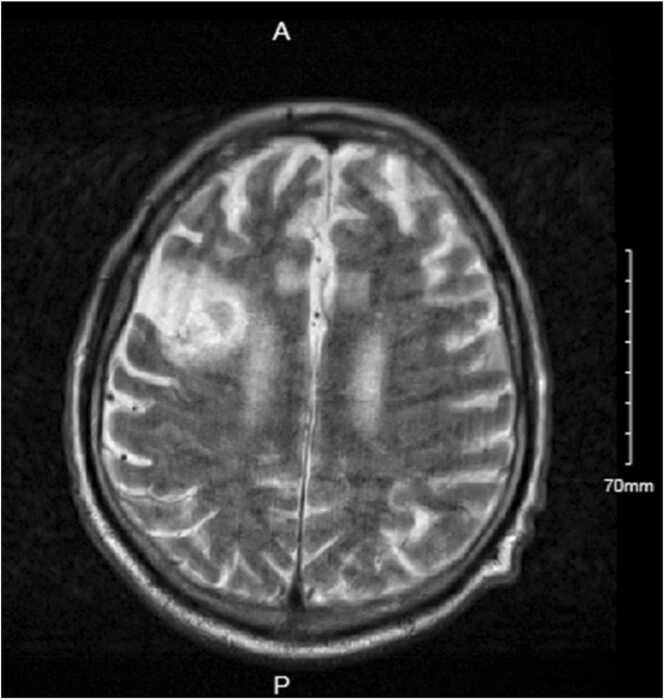
MRI Brain demonstrating a 6 mm ring enhancing lesion concerning for mycotic aneurysm.

Clinically, he remained haemodynamically stable. His discoloured, grey fingers were reviewed by the Rheumatology team and determined to be more consistent with atypical Raynaud’s than with vascular phenomenon of IE. Cardiothoracic surgeons assessed the patient for operative intervention. However, surgical timing was complicated by new magnetic resonance imaging (MRI) brain demonstrated a 6 mm right rim enhancing lesion at the right sylvian fissure (Fig. 2), subarachnoid haemorrhage, and infarct of unclear age. The patient was reviewed by the Neurology, who determined the ischaemia to be old in nature, and did not object to operative management of IE.

**Fig. 3:**
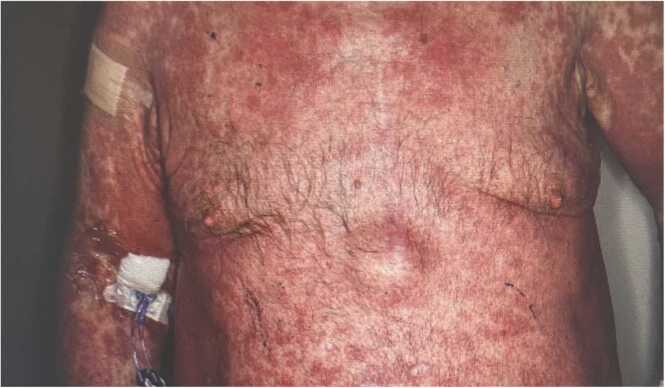
diffuse, erythematous morbilliform rash due to DRESS.

The patient’s course was further complicated by a diffuse, erythematous, morbilliform rash involving the face, trunk, and extremities on day 26 into admission. This occurred three weeks after starting ceftriaxone. The rash also developed seven-eight days after the reintroduction of vancomycin, restarted due to rising CRP. In the days preceding the rash, the patient’s eosinophil count was mildly elevated at 0.53 (reference range; 0–0.5 ×10⁹/L). Gamma-glutamyl transferase was elevated at 114 IU/L (reference range; 11–67 IU/L), as were alkaline phosphatase levels at 283 IU/L (reference range: 30–130 IU/L). Dermatology performed a skin biopsy, which showed vacuolar interface dermatitis, consistent with Drug Reaction with Eosinophilia and Systemic Symptoms (DRESS) (Fig. 3). The reaction led to the discontinuation of vancomycin and ceftriaxone. The antibiotics were held for one week and once his rash improved with topical emollients, he was switched to amoxicillin.

**Fig. 4:**
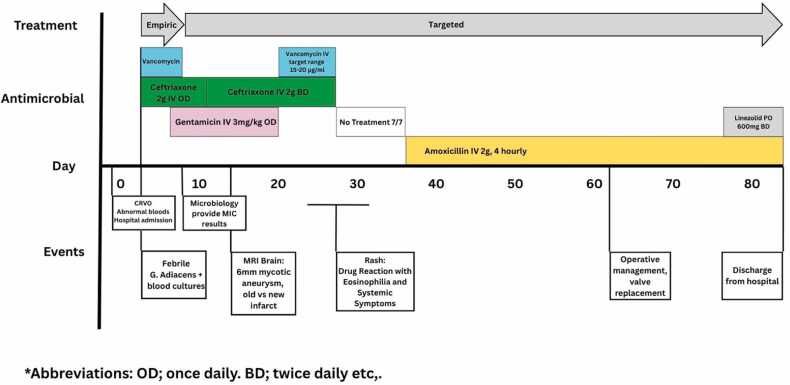
*Timeline of antimicrobial administration periods and clinical events*.

Surgical timing was further complicated by microangiopathy, as the principal driver of the patient’s thrombocytopenia and anaemia, and subacute bacterial endocarditis-related glomerulonephritis. It was decided that any operative intervention would be deferred until the patient’s platelet count exceeded 100 × 10⁹/L and until his haemoglobin level rose above 8 g/dL. In the interim, antimicrobial coverage was extended until surgical intervention could be safely performed. At that time, repeat TOE showed that the vegetation remained unchanged and clinically the patient continued to experience intermittent fevers, despite having completed 42 days of intravenous antibiotic therapy.

Finally, on day 62 of admission (Day 59 of antimicrobial therapy), the patient’s platelets and haemoglobin were considered adequate for operative management. He underwent a successful redo aortic valve replacement with a 21 mm Resilia tissue valve. Intra-operative valve tissue histology showed hyalinised fibroconnective tissue with dystrophic calcification and no vegetations observed. The heart valve cultures were no growth. The patient made a good recovery post-operatively. He still received IV amoxicillin for three weeks post-operatively and linezolid was introduced shortly prior to discharge. This was due to multiple undrained splenic abscesses. The patient was eventually discharged after 85 days of hospital admission.

**Patient Perspective**: The patient understood the seriousness of the diagnosis, having initially sought care solely from his optician. Nevertheless, he accepted the unexpected diagnosis and expressed gratitude for the professionalism and care provided.

## Discussion

The indolent nature of *G. adiacens* IE poses diagnostic challenges and contributes to an increased morbidity and mortality [2], [3], [4]. Unlike more virulent organisms, *G. adiacens* tends to cause a less aggressive clinical presentation [4]. This, combined with its complex nutritional requirements, make it a challenging organism to identify and culture [1]. Similarly to our case, it has been reported that patients often present with non-specific symptoms and do not seek treatment until significant complications occur [2], [3]. Our patient differed from other case reports in that the retinal findings that led to admission were completely incidental and based solely on a routine optician checkup.

This case also highlights the importance of recognising associations between microorganisms and specific clinical syndromes. The identification of *G. adiacens* in blood cultures should prompt clinicians to investigate for IE, particularly in patients with major risk factors such as valvular heart disease, congenital heart defects, prosthetic valves, or a history of intravenous drug use [1], [4]. Although nutritionally variant streptococci (NVS) are responsible for only approximately 5 % of IE cases [1]—with *G. adiacens* accounting for an even smaller proportion—they are associated with a higher rate of complications and greater antibiotic resistance compared to more common organisms such as *viridans* streptococci [1], [3], [5].

This case also demonstrates the potential of *G. adiacens* to manifest extracardiac features of IE. Extracardiac features can include rare presentations such as diffuse alveolar haemorrhage or infection-related glomerulonephritis, as seen in our patient [8]. Previous studies have suggested that NVS IE is associated with an increased risk of extracardiac vascular complications such as septic emboli and mycotic aneurysms [2], [6], [7]. Although this could in part be related to the delay in diagnosis typically associated with IE secondary to these organisms. *G. adiacens* has also been implicated in infections related to cardiac implantable electronic devices, which carry the same management challenges [10], [11].

A factor contributing to the difficulty in diagnosing cases such as this, is the fastidious nature of the organism, which has specific growth requirements. These include the need for vitamin B6 analogues and L-cysteine in the growth medium, both of which are essential for successful cultivation and identification [1]. Fortunately, in our case, blood cultures were positive for *G. adiacens* within days of admission, allowing for prompt and targeted diagnostic management. However, this is often not the case in similar patient cohorts, where delayed culture growth can hinder early diagnosis and treatment [2], [3], [5].

A notable challenge in managing *G. adiacens* infections is the absence of EUCAST-defined antimicrobial susceptibility breakpoints for this organism [1]. As a result, microbiology reports typically provide minimum inhibitory concentrations (MICs) without interpretive categories such as susceptible (S), intermediate (I), or resistant (R). This can create uncertainty in clinical decision-making. In such cases, clinicians may refer to the Clinical and Laboratory Standards Institute (CLSI) guidelines, which do include breakpoints for nutritionally variant streptococci, including *G. adiacens* [9]. While extrapolation from CLSI standards can help guide therapy, treatment decisions should also consider the clinical context, antibiotic pharmacokinetics/pharmacodynamics, and ID specialist input.

Finally, a significant challenge in our patient’s case was the timing of surgical intervention. The risk of embolism and death is closely linked to echocardiographic findings such as vegetation size and mobility [12]. While early surgery is recommended by both the European Society of Cardiology (ESC) and the American Heart Association/American College of Cardiology (AHA/ACC) for patients with complicated IE, there is limited data published to guide physicians [6]. Our patient met the ESC criteria for uncontrolled infection, another indicator favoring early surgery in IE [6]. The ESC defines uncontrolled infection as “either abscess, fistula, or pseudoaneurysm; or for an enlarging vegetation, persistent fever, or positive blood cultures after 7–10 days of appropriate therapy.”

The ESC also recommends urgent surgery in cases where a vegetation > 10 mm is present with evidence of embolisation [6], [7], as was the case in our patient. Current guidelines also support surgical intervention for patients with prosthetic valve endocarditis (PVE) given its increased risk of poorer outcomes and higher rates of treatment failure with antibiotics alone [6]. However, early surgery poses a significant risk for patients with neurological complications of IE, such as mycotic aneurysm and stroke [6]. This is mostly due to the increased bleeding risks associated with the use of anticoagulation during cardiopulmonary bypass, but also may be secondary to thrombocytopenia, which was the case for our patient. However, the risk of poor post operative outcomes must be weighed against the risk of further cardiac deterioration from postponing surgery. It has been suggested that delaying surgery in patients with neurological complications led to worse outcomes overall for these patients [6]. The ESC guidelines support early surgery in patients who have experienced mild ischaemic cerebral events but advise postponing surgery for those with cerebral haemorrhage or severe stroke [6]. Ultimately, it is a decision that needs to be made on a case-by-case basis with significant multidisciplinary involvement.

In summary, this case was discovered incidentally after a central retinal vein occlusion and despite minimal symptoms, he had aortic valve vegetation with multiple embolic complications and delayed surgical intervention due to dermatological, renal and haematological issues; highlighting the subtle presentation and complexity of managing *G. adiacens* PVE.

## Author contributions

Each author has made substantial contributions to the conception, design, acquisition, analysis, or interpretation of data and has participated in drafting or critically revising the manuscript. All authors have approved the final version of the manuscript and agree to be accountable for all aspects of the work.

## Originality and exclusivity

This manuscript is original and has not been published or submitted elsewhere. It is not under consideration by another journal or publisher.

## Copyright assignment / licensing

The authors agree to transfer copyright (or grant a license, depending on journal policy) to the publisher upon acceptance, as per the journal's guidelines.

## Corresponding author responsibility

The corresponding author will act on behalf of all co-authors in all matters related to the manuscript submission and publication process.

## CRediT authorship contribution statement

**Daniel Broderick:** Methodology, Writing – original draft, Writing – review & editing. **Carlos Mejia-Chew:** Conceptualization, Investigation, Methodology, Project administration, Resources, Supervision, Writing – original draft, Writing – review & editing. **Varisha Shahzad:** Investigation. **Yvonne O’Meara:** Writing – review & editing. **James McCarthy:** Writing – review & editing. **Emer Kilbride:** Writing – review & editing. **James Woo:** Writing – review & editing. **Padraig McGettrick:** Writing – review & editing. **Ciara Murray:** Investigation, Methodology, Writing – original draft.

## Patient consent

Informed consent was obtained from the patient for publication of this case report and all accompanying images. A copy of the patient consent is available for review upon request.

## Ethical approval

Where human subjects are involved, all necessary ethical approvals and patient consents have been obtained and documented in accordance with institutional and international standards.

## Funding

This research did not receive any specific grant from funding agencies in the public, commercial, or not-for-profit sectors.

## Declaration of Competing Interest

All potential conflicts of interest, financial or otherwise, have been disclosed. Where applicable, disclosures are included within the manuscript.
